# Effect of availability of HIV self-testing on HIV testing frequency among men who have sex with men attending university in China (UniTest): protocol of a stepped-wedge randomized controlled trial

**DOI:** 10.1186/s12879-020-4807-4

**Published:** 2020-02-18

**Authors:** Song Fan, Zhongquan Liu, Zhenzhou Luo, Maohe Yu, Lin Ouyang, Hui Gong, Yi Ding, Peiyang Li, Tanwei Yuan, Yepeng Zhou, Guohui Wu, Huachun Zou

**Affiliations:** 1 School of Public Health, Sun Yat-sen University, Guangzhou, 510080 Guangdong China; 2 School of Public Health, Southwest Medical University, Luzhou, 646000 Sichuan China; 3 Department of AIDS/STD Control and Prevention, Tianjin Center for Disease Control and Prevention, Tianjin, 300011 China; 4 Nanshan District Center for Chronic Disease Control, Shenzhen, 518000 China; 5 Department of AIDS/STD Control and Prevention, Chongqing Center for Disease Control and Prevention, Chongqing, 400042 China; 6 School of Public Health (Shenzhen), Sun Yat-sen University, Shenzhen, 510006 Guangdong China; 7 Foshan Pengyou AIDS Prevention Care Center, Foshan, 528000 Guangdong China; 8 Kirby Institute, University of New South Wales, Sydney, Australia; 9 School of Public Health, Shanghai Jiao Tong University, School of Medicine, Shanghai, China

**Keywords:** HIV self-testing, Young men who have sex with men, Student, Stepped-wedge design

## Abstract

**Background:**

HIV testing plays a central role in the combat against HIV. We aimed to determine if the availability of HIV self-testing (HIVST) would increase the frequency of testing among men who have sex with men (MSM) attending university in China.

**Methods:**

A stepped wedge randomized controlled trial will be conducted in 4 provinces in China: Chongqing, Guangdong, Shandong, and Tianjin. Eligibility assessment will include (1) male, aged 16 years or older, (2) university student (technical diploma and undergraduate students), (3) MSM (sexual behaviors including mutual masturbation, oral sex, and anal sex), (4) HIV negative, and (5) willing to provide informed consent.

Participants will be randomly allocated to HIV self-testing intervention with free HIVST kits in every 30 days according to the intervention waiting lists with a computer-generated randomized sequence. All participants will complete a self-administrated online questionnaire onsite at baseline and 12-month follow-up and complete an online questionnaire at 4- and 8-month.

The primary outcome is the effect of HIVST on HIV testing frequency. Secondary outcomes include the change in sexual behaviors and HIV incidence.

**Discussion:**

No previous study had measured the effect of social media based HIVST intervention on the change in HIV testing behaviors, sexual behaviors and incident HIV infection among MSM attending university in China. Findings from this study will provide evidence for further interventional practice promotions and prevention strategies scale-up, including HIV testing, pre-exposure prophylaxis (PrEP) or post-exposure prophylaxis (PEP), and sexual partner serosorting.

**Trial registration:**

Chinese Clinical Trial Registry: ChiCTR1900020645. Registered 11 January 2019.

## Background

University students in China are facing increasing risk of human immunodeficiency virus (HIV) transmission [1]. According to the National Center for AIDS/STD Control and Prevention (NCAIDS), from 2011 to 2015, the number of university students infected with HIV increased by 35% annually [2]. The five years (2013–2017) surveillance reported 12,037 newly diagnosed student HIV cases, 97.7% were male, and 82.2% were men who have sex with men (MSM) [3]. MSM attending university refers to male university students who have oral or anal sex with other men, regardless of their sexual orientation or sexual identity. Systematic reviews found that the HIV prevalence among this population was 3.8% [4] to 4.4% [5]. Studies found high (65.2%) unprotected anal intercourse (UAI) in the past 6 months and low (57.5%) condom use in last anal intercourse among them [4].

Since the Joint United Nations Program on HIV/AIDS (UNAIDS) set up the ‘90–90-90’ goal to end the HIV epidemic by 2030, HIV testing has been placed in the forefront to ensure early detection and treatment of HIV case [6]. China has adopted an active approach of HIV testing in high-risk groups such as injecting drug users (IDUs), sex workers and MSM since 2004 [7]. In 2014, 1881 HIV sentinel surveillance sites completed the HIV testing of 750, 000 people in China [8]. Although HIV testing coverage has been steadily increasing, only half of MSM had ever tested for HIV [9, 10]. Stigma, discrimination, and lack of privacy are barriers for MSM to access HIV testing [11, 12]. HIV self-testing (HIVST) is a process that a person takes and interprets a test, which is recommended by the World Health Organization (WHO) as an acceptable, private, and convenient approach to HIV testing [13, 14]. Currently only one urine-based HIV self-test was officially approved by health authorities in China [15]. Some studies reported that between 29% and 40% of participants had conducted HIV self-testing among Chinese MSM [16, 17]. An internet-based HIVST study suggested HIVST can promote active testing, eliminate the concern of privacy disclosure, and improve the accessibility and pertinence of HIV testing among MSM in China [18]. According to *The China Action Plan for the Thirteenth Five-Year Plan for Combating and Prevention of AIDS* which was issued by the State Council of China, self-testing should be applied to scale up HIV testing [19].

MSM attending university are a unique subgroup of the MSM community. Understanding the key factors of HIV transmission among this population is crucial to providing sufficient support and services. HIV prevention among MSM attending university will largely depend on interventions that can reduce unprotected sex and increase the frequency of HIV testing. In recent years, with the increasing promotion of HIV testing within the MSM community, more MSM started to access HIV and other sexually transmitted infections (STIs) testing services, supposedly increasing the frequency of testing among students. Despite these efforts, large numbers of students living with HIV are unaware of their status, increasing chances of immune deterioration and secondary transmission [20–23]. Compared to general MSM, there are additional barriers to HIV testing among students, including low-risk perception, poor knowledge and understanding, fear of positive diagnosis, concern of disclosure of sexuality, and limited access and testing resources [24]. HIVST may be a potential approach to increase access to HIV testing for students who avoid in-person HIV testing sites due to perceived challenges to confidentiality [25, 26].

HIV-related research among MSM attending university in China is limited by low representativeness (i.e., samples were a small fraction of general MSM). Within China, prevention efforts for MSM attending university are significantly weaker in evidence base than those for other high-risk groups. The limited data generated by cross-sectional studies to date cannot provide accurate estimates of HIV infection and characterize the factors driving transmission among MSM attending university in China, which further inhibits the development of effective interventions for this population. Prospective surveys among this population will be more helpful in understanding the risks for HIV infection and the effectiveness of interventions among MSM attending university. Effective intervention approach (i.e., availability of HIV self-testing) which can facilitate HIV diagnosis and prevent secondary transmissions also needed to be developed and evaluated using rigorous methodologies.

## Methods/design

### Study aims

The University Student HIV Test Intervention Study (the UniTest Study) aims to conduct a randomized controlled trial (RCT) with a stepped-wedge design to evaluate the effect of HIVST intervention on HIV testing frequency, HIV related high-risk behaviors and HIV incidence among MSM students attending university in China.

### Study design

We will use a stepped wedge trial (SWT) design to implement HIV self-testing intervention. Participants will randomly receive HIVST intervention at different time points. Unlike the traditional parallel designed trials, in an SWT participants are randomly allocated to the order of implementation, such that all participants eventually receive the intervention [27, 28]. The SWT design is recommended for trials that the intervention will do better than harm [29], such as HIVST intervention. Compared to parallel design, the SWT design maximizes statistic power and needs fewer clusters [30]. In our study, we will gradually implement HIVST intervention every 30 days according to the intervention waiting lists with a computer-generated randomized sequence.

### Study population

#### Inclusion criteria

Individuals are eligible if they meet the following criteria: 1) Male, aged 16 years or older. 2) University student (technical diploma and undergraduate students). 3) MSM (sexual behaviors including mutual masturbation, oral sex, and anal sex). 4) HIV negative. 5) Willing to provide informed consent.

### Sample size

The sample size is calculated based on the HIV testing rate of MSM. A systematic review showed that just over 40% of MSM attending university in China had HIV testing in lifetime [9]. The HIV testing rate of the control group is estimated to be 40.0% (*p*_0_ = 0.40); Anticipated HIV testing rate of the intervention group will increase to 60% (*p*_1_ = 0.60), a total of six clusters, six total intervention time periods, a coefficient of variation of 0.4, two-sided *α* = 0.05, and 90% power. The annual loss to follow-up rate of the general MSM is 30.0%. The sample size is 408 (68 for each cluster). To further improve the power for sub-analysis, we increased the sample size by 10% to 448 (112 for each region) (see Additional file 1) [27].

### Study setting

The UniTest Study will conduct in 4 provinces based on geographical location and distribution of colleges and universities (Table 1).

**Table 1:** Study setting of the UniTest Study

Area	Location	CDC/CBOs	Sample size
Southern	Guangdong province	Lingnan parners Pengyou care Nanshan Center for Chronic Disease Control	112
Western	Chongqing municipality	Chongqing CDC	112
Northern	Tianjin municipality	Tianjin CDC Shenlan	112
Eastern	Shandong province	Qingai	112

*CDC* Center for disease control

*CBOs* Community-based organizations

**Fig. 1:**
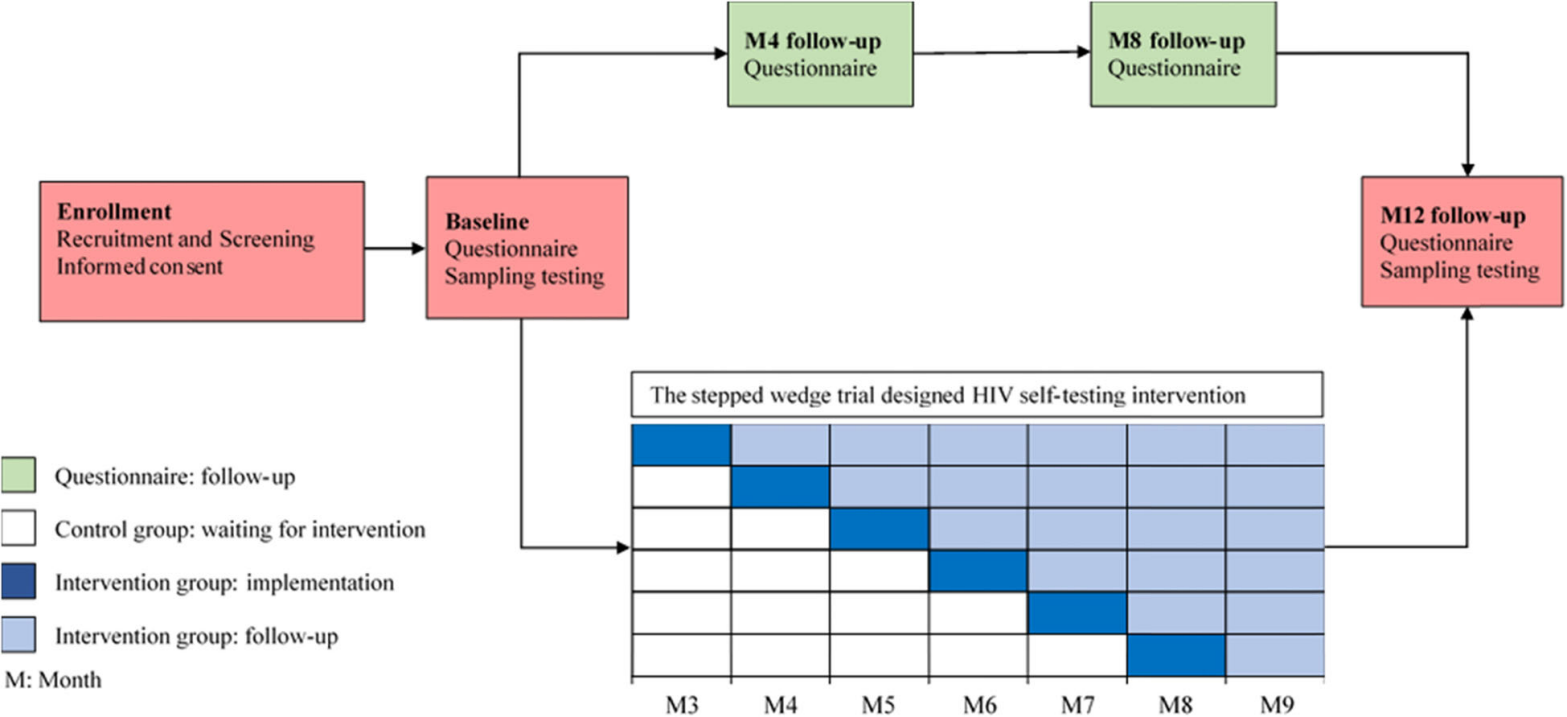
The flow chart of the UniTest study. Enrollment procedure: This section includes recruitment, screening, informed consent, and registration. Baseline survey procedure: This section includes a baseline questionnaire and sampling testing. Follow-up survey and intervention procedure: This section is divided into two parallel parts, including questionnaire follow-up and the stepped wedge trial designed HIV self-testing intervention. End-point survey procedure: This section includes 12 months follow-up questionnaire and sampling testing

The UniTest Study began in November 2018 and is scheduled to complete by December 2020. The study has four study phases, including enrollment, baseline survey, follow-up survey and intervention, and 12 months end-point survey (Fig. 1).

### Enrollment procedure

Participants recruitment will be promoted both online and offline. For online: study advertising campaigns will be placed on the social network such as WeChat [31, 32] (WeChat is a Chinese multi-purpose messaging, social media and mobile payment app developed by Tencent Inc., Shenzhen, China.) and gay community web pages. For offline: the study advertisement will be distributed with the collaboration of community-based organizations (CBOs), health clinics, and student associations. A WeChat account will be established solely for online consultation and follow-up. After reading study advertisement online or offline, potential participants can learn more about the study through the contact information on the advertisement and make an appointment to join the study at a preferred CBO.

Participants will complete the screening procedure and receive a rapid test at a given CBO. An online registration page will guide each participant to complete the sign-up process, including the UniTest Study introduction, eligibility assessment, informed consent, study identification (ID) number assignment, and mobile phone number verification.

### Questionnaire survey

We will use a self-administrated online questionnaire to record the information of participants. After scanning the Quick Response (QR) code by mobile phone, participants can verify their identities and fill in the questionnaire. The questionnaire contains different structured modules with the behavioral intervention theory design (Table 2). Participants will complete a self-administrated online questionnaire onsite at baseline and 12-month follow-up and complete an online questionnaire at 4- and 8-month.

**Table 2:** Summary of variables collected at baseline and follow-up visits

Variable modules	Time point(s)
Personal verification information	Baseline
Socio-demographic information	Baseline
Sexual behavior information	Baseline/Follow-up
Social media use behaviors	Baseline/Follow-up
Self-cognition scale	Baseline
HIV infection susceptibility/severity perception scale	Baseline/Follow-up
HIV testing obstacle perception scale	Baseline/Follow-up
HIV testing benefit perception scale	Baseline/Follow-up
HIV testing self-efficacy scale	Baseline/Follow-up
HIV testing behaviors	Baseline/Follow-up
Others: STIs, alcohol use, drug use	Baseline/Follow-up

### Sampling and testing

We will collect blood specimen and rectal swab specimen on-site at baseline and 12-month follow-up visits. Trained doctors will collect 10 ml blood specimen and conduct HIV rapid testing. Participants will self-collect a rectal swab guided by a self-collection instruction (see Additional file 2) [33].

Specimens will be labelled with unique survey ID numbers that match the participant’s laboratory requisition form, questionnaire, and results form. No personally identifying information will be included on any specimen, or laboratory form.

Blood specimens will be tested for hepatitis A (HAV), hepatitis B (HBV), and hepatitis C (HCV) in the certified laboratory (Hybribio Medical Laboratory, Guangzhou, China). The HAV, HBV, and HCV status will be determined by Enzyme-linked immunosorbent assay (ELISA) (Autobio Biological engineering co., Ltd., Zhengzhou, China).

Rectal swab specimens will be collected to test for thirty-seven subtypes of human papilloma virus (HPV), including HPV6, 11, 16, 18, 26, 31, 33, 34, 35, 39, 40, 42, 43, 44, 45, 51, 52, 53, 54, 55, 56, 57, 58, 59, 61, 66, 67, 68, 69, 70, 71, 72, 73, 82, 83, 84, CP8304. HPV status will be determined by Polymerase chain reaction (PCR) (Hybribio Biochemical co., Ltd., Chaozhou, China).

### HIV testing

The HIV testing will be conducted with rapid HIV testing (Alere® Determine HIV-1/2, Alere Medical Co., Ltd., Matsudo, Japan) at baseline and 12-month follow-up. Participants tested HIV negative result will be included in the cohort. Participants tested positive for HIV will be referred to local centers for disease control and prevention for confirmation testing and subsequent services as per guidelines (*Regulations on the Prevention and Control of AIDS)* [34].

### Intervention process

**Fig. 2:**
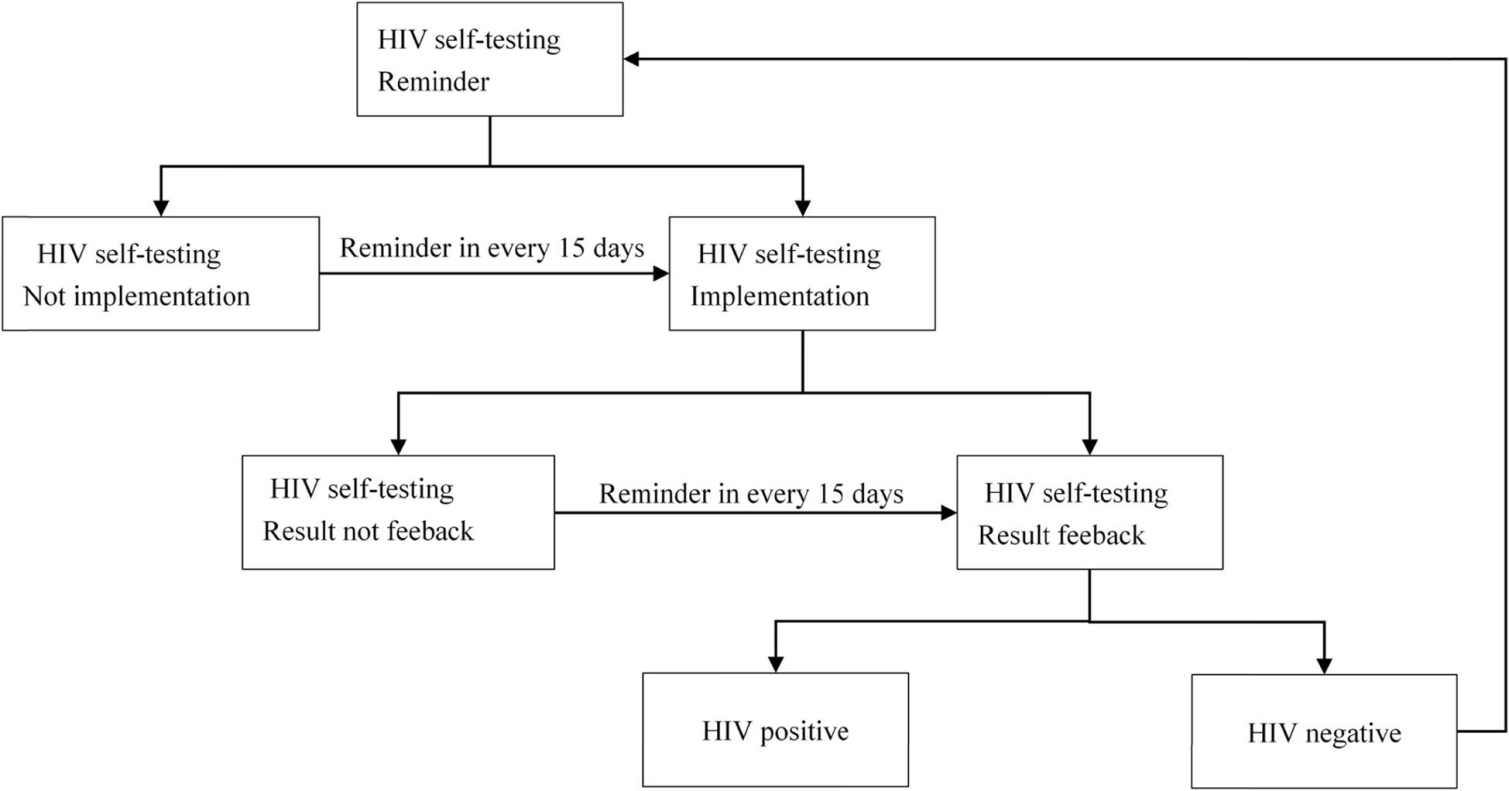
The flow chart of the HIV self-testing intervention

HIVST intervention process will last for six months, including HIV testing reminders, HIVST kit distribution, and HIVST result feedback (Fig. 2).

#### Intervention group

Once allocated to the intervention group, participants will be provided HIV self-testing comprehensive Intervention, including HIVST kits with testing and feedback reminders. We encourage our participants to perform HIV tests for themselves and their sexual partners with HIVST kits. At each intervention, participants will be provided two HIVST kits along with instructions. Each participant will receive up to two deliveries of a total of four HIVST kits during 6 months intervention period.

#### HIV self-testing kit

A finger-prick-based HIV self-testing package contains an HIV testing kit (Alere® Determine HIV-1/2, Alere Medical Co., Ltd., Matsudo, Japan), band-aid, alcohol pad, disposable peripheral blood needle, and operation instruction card. The HIVST kit was a third-generation rapid reagent approved by the US Food and Drug Administration (FDA) and China State Food and Drug Administration (CSFDA), it can detect HIV 1/2 antibodies in blood specimens at six weeks post-infection. The specificity and sensitivity of the Alere® Determine HIV 1/2 are 99.75 and 100%, respectively. The testing result can be read in 15–60 min [35].

#### HIVST kit distribution

A reminder to test for HIV will be sent to a participant via WeChat on the first day of enrollment. This opt-out reminder will be sent at 15-day intervals until participants apply for the HIVST kit. After receiving the message, the participant can click the link to fill out information for applying for HIVST kit. HIVST kits will be delivered anonymously and confidentially via post express.

#### HIVST result feedback

Participants will be guided by either the printed instruction included in the kit package or an online demonstration video by scanning the QR code, to complete self-testing procedures.

After finishing self-testing, participants can upload the testing results via the online feedback system. The system will record the test results filled in by the participants themselves; then research staff will conduct a doubled check for the results.

A feedback reminder will send a message to the participant after confirming receiving HIVST kit. If the participant has not uploaded his testing result, the feedback reminder will repeatedly send text in every 15 days.

### Control group

Participants who are waiting for randomized allocation of intervention, are treated as control group that will not receive HIVST intervention. These men will need to complete a questionnaire at 4- and 8-months.

### Study endpoint

1. HIV positive: Participants who have positive results with HIVST will be provided with further consultation for the testing results and recommendations to local CDC for confirmatory laboratory testing. If the participants are laboratory confirmed HIV positive, the study process (follow-up and intervention) will be discontinued.
2. Loss to follow-up.
3. Close-out of study: Participants finish 12-month follow-up and testing.

### Outcome measures

The primary outcome is the change in frequency of HIV testing comparing before and after intervention. Secondary outcomes include the change in sexual behaviors comparing before and after intervention, and HIV incidence comparing before and after intervention (Table 3).

**Table 3:** Key indicators of the UniTest Study

Indicators		Definition
HIV testing Behavior	HIV testing in the control group	The frequency of HIV testing before receiving the intervention
	HIV testing in the intervention group	The frequency of HIV testing after receiving the intervention
	HIV testing reminders	The frequency of sending HIV testing reminders during the study period
	HIV self-testing kit application	The frequency of self-testing kits applications during the study period
	HIV self-testing result feedback	The frequency of self-test results feedback during the study period
Time indicator	Control time	The time interval of the participants to wait for receiving the intervention
	Intervention time	The time interval of the participants from receiving the intervention to the endpoint
HIV testing result	HIV infections	HIV infections detected during the study period
	HIV infections in the control group	HIV-positive individuals tested before intervention
	HIV infections in the intervention group	HIV-positive individuals tested after intervention

### Data management and statistical analysis

All data will be recorded directly in the online database (www.wjx.cn, Ranxing information technology co., Ltd., Changsha, China). The database system automatically saves the data and ensures the security, integrity, and consistency of the data. The network server is hosted in Alibaba Cloud (Alibaba Group, Hangzhou, China) and installed with enterprise firewall protection, while the daily backup mechanism ensures data security. The data have multiple levels of permission settings and password protection to ensure data security.

In the statistical analysis, the general statistical analysis will be carried out by using standard statistical software and professional statistical software. If more advanced statistical methods are needed to complete the data processing and utilization, the training of data analysis and utilization can be added.

### Descriptive statistical analysis

The analysis will be carried out for all variables and outcomes involved in the study according to the types of variables, including count, frequency, ratio, mean, standard deviation, and so on.

### Latent variable analysis and structural equation model

We will conduct a latent variable analysis to explore the indicators and connected potential variables. After the latent variable analysis, the path analysis diagram will show the relationship between potential variables and explicit variables in the structural equation model. We will use structural equation model (SEM) to evaluate the effect of HIVST intervention, estimate the association among behavioral intervention theory modules (such as social demographic characteristics, social cognition, social norms, susceptibility to perceived HIVST benefits, self-efficacy, etc.), sexual behavior, HIV testing behavior, HIV infection, and HIVST. Meanwhile, multiple model fitting and comparison will be conducted to select the best model that can explain the effect and influencing factors of HIVST intervention among MSM attending university in China.

### Missing data

We will record the reasons for missing data. Multiple imputation (MI) methods will be conducted to increase the robustness of study results.

### Ethics and dissemination

Participation in the UniTest Study is anonymous and voluntary. The study has been approved by the ethics review board of Sun Yat-sen University (SYSU-SPH2018044) in China. Participants will benefit from frequent testing and early diagnosis of HIV and timely treatment if positive. We anticipate that the benefits of study participation will outweigh any risks, including disclosure of sexual behaviors. Our findings will be disseminated to the local and national government in China as well as the broader academic audience via peer-reviewed publications and academic conferences.

## Discussion

The UniTest Study will conduct HIV self-testing intervention with stepped-wedge design randomized controlled trial among MSM attending university. So far, to the best of our knowledge, this is the first prospective study to implement HIVST intervention and measure its effect on HIV testing and sexual behaviors with the application of social media among this population in China. We expect that HIVST will provide opportunities for further intervention (HIV testing) scale-up, prevention synergies with pre-exposure prophylaxis (PrEP) or post-exposure prophylaxis (PEP) (which requires frequent testing), and harm reduction strategies (sexual partner serosorting).

This study also faces potential challenges. First, recruiting enough qualified participants is difficult. There are still obstacles to out-for HIV testing among students, including fear of positive diagnosis, the concern of disclosure of sexuality, and limited access to resources [24]. To recruit more participants, we will try multiple approaches, including peer recommendations, social media, gay apps, and campus advertising. Second, there are time intervals while waiting for intervention and follow-up. It takes considerable commitment to avoid loss to follow-up. Participants will be asked to verify their mobile phone number and connect with WeChat study account at the enrollment process. Meanwhile, the WeChat account will regularly send health education information, various types of activity information, and incentive information to increase compliance and reduce the loss of follow-up. Third, it remains unknown what proportion of individuals who find a positive result will continue reporting and seeking for confirmation. In the UniTest Study, we will make sure that participant with HIVST positive result will be referred to the CDC for confirming testing and further service. For participants who do not provide HIVST result feedback, we will continue to send reminders in every 15 days for 3 months and request the local CBOs to recontact.

We believe that the lessons learned from the UniTest Study will guide practice decision on HIVST promotion among MSM attending university. The results will also contribute to comprehensive, evidence-based recommendations on policies, practices, and strategies to reduce the risk of HIV infection and promote health among this young population. Moreover, knowledge gained on the potential benefit of HIVST intervention may apply to other vital groups for AIDS prevention and control.

### Trial status

**Fig. 3:**
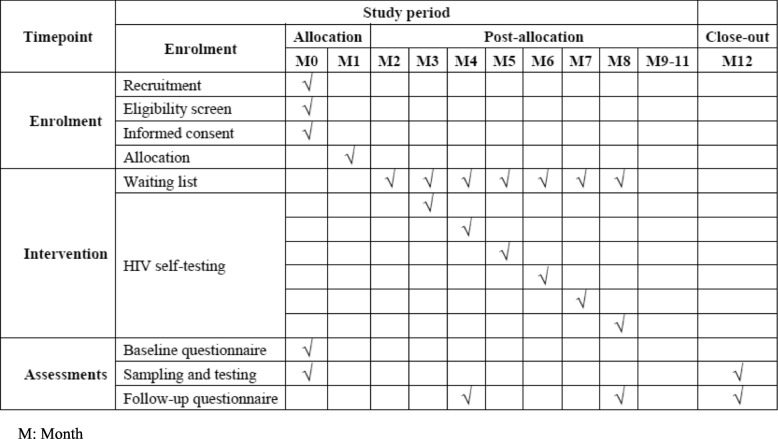
Schematic diagram for the schedule of enrolment, interventions, and assessments

Enrolling and data collection. The trial protocol conforms to the Standard Protocol Items: Recommendation for Interventional Trials (SPIRIT) 2013 statement [36] (Fig. 3, Additional file 3).

## Supplementary information

**Additional file 1.** Table for sample size calculation.

**Additional file 2.** Self-collection instructions for rectal swab specimen collection.

**Additional file 3.** SPIRIT Checklist.

## Data Availability

The data collected in this study will not be publicly available. However, the corresponding author can be contacted for de-identified data on reasonable request.

## Abbreviations

CBOs: Community-based organizations
CDC: Center for disease control
CSFDA: China State Food and Drug Administration
ELISA: Enzyme-linked immunosorbent assay
FDA: US Food and Drug Administration
HAV: Hepatitis A
HBV: Hepatitis B
HCV: Hepatitis C
HIV: Human immunodeficiency virus
HIVST: HIV self-testing
HPV: Human papilloma virus
ID: Identification
IDUs: Injecting drug users
MI: Multiple imputation
MSM: Men who have sex with men
NCAIDS: National Center for AIDS/STD Control and Prevention
PCR: Polymerase chain reaction
PEP: Post-exposure prophylaxis
PrEP: Pre-exposure prophylaxis
QR: Quick Response
RCT: Random control trial
STIs: Sexually transmitted infections
SWT: Stepped wedge trial
SYSU: Sun Yat-sen University
UAI: Unprotected anal intercourse
UNAIDS: The Joint United Nations Program on HIV/AIDS
UniTest Study: University Student HIV Test Intervention Study
WHO: World Health Organization
