# Supplementary material for: Effect of availability of HIV self-testing on HIV testing frequency among men who have sex with men attending university in China (UniTest): protocol of a stepped-wedge randomized controlled trial
Source: BMC Infect Dis. 2020 Feb 18;20:149. doi: 10.1186/s12879-020-4807-4 (PMC7029612; doi:10.1186/s12879-020-4807-4)
Supplement: Supplementary file 1 — Additional file 1. Table for sample size calculation. [file 12879_2020_4807_MOESM1_ESM.docx]

Supplementary file 1.

**Table for sample size calculation**

| ***p*_c_** | ***p*_i_** | **Number of clusters** | **Number of time periods** | **Coefficient of variation** | ***α*** | **Power** | **Sample size**  **(no LTFU)** | **LTFU** | **Sample size for each cluster** | **Total sample size**  **(6 clusters)** |
| --- | --- | --- | --- | --- | --- | --- | --- | --- | --- | --- |
| 30 | 40 | 6 | 6 | 0.4 | 0.05 | 0.9 | 80 | 30% | 104 | 624 |
| 30 | 40 | 6 | 6 | 0.15 | 0.05 | 0.9 | 68 | 30% | 88 | 528 |
| 30 | 50 | 6 | 6 | 0.4 | 0.05 | 0.9 | 20 | 30% | 26 | 156 |
| 30 | 50 | 6 | 6 | 0.15 | 0.05 | 0.9 | 14 | 30% | 18 | 108 |
| 30 | 60 | 6 | 6 | 0.4 | 0.05 | 0.9 | 8 | 30% | 10 | 60 |
| 30 | 60 | 6 | 6 | 0.15 | 0.05 | 0.9 | 6 | 30% | 8 | 48 |
| 40 | 50 | 6 | 6 | 0.4 | 0.05 | 0.9 | 88 | 30% | 114 | 684 |
| 40 | 50 | 6 | 6 | 0.15 | 0.05 | 0.9 | 80 | 30% | 104 | 624 |
| **40** | **60** | **6** | **6** | **0.4** | **0.05** | **0.9** | **52** | **30%** | **68** | **408** |
| 40 | 60 | 6 | 6 | 0.15 | 0.05 | 0.9 | 28 | 30% | 36 | 216 |
| 50 | 60 | 6 | 6 | 0.4 | 0.05 | 0.9 | 89 | 30% | 116 | 696 |
| 50 | 60 | 6 | 6 | 0.15 | 0.05 | 0.9 | 83 | 30% | 108 | 648 |

Note: *p*_c_: probability of HIV testing during control period; *p*_i_: probability of HIV testing during intervention period;

LTFU: Loss to follow-up
