# Supplementary material for: Effect of availability of HIV self-testing on HIV testing frequency among men who have sex with men attending university in China (UniTest): protocol of a stepped-wedge randomized controlled trial
Source: BMC Infect Dis. 2020 Feb 18;20:149. doi: 10.1186/s12879-020-4807-4 (PMC7029612; doi:10.1186/s12879-020-4807-4)
Supplement: Supplementary file 2 — Additional file 2. Self-collection instructions for rectal swab specimen collection. [file 12879_2020_4807_MOESM2_ESM.docx]

Supplementary file 2

**Self-collection instructions for rectal swab specimen collection**

1. Partially peel open the swab package. Remove the swab. Do not touch the soft tip or lay the swab down. If the soft tip is touched, the swab is laid down, or the swab is dropped, use a new Swab Specimen Collection Kit.
2. Hold the swab, placing thumb and forefinger in the middle of the swab shaft covering the score line. Do not hold the swab shaft below the score line.
3. Carefully insert the swab into the rectum about 1-2 inches (3-5 cm) past the anal margin and gently rotate the swab for 5 to 10 seconds. Withdraw the swab without touching the skin.
4. While holding the swab in the same hand, unscrew the cap from the tube. Do not spill the contents of the tube. If the contents of the tube are spilled, use a new Swab Specimen Collection Kit.
5. Immediately place the swab into the transport tube so that the score line is at the top of the tube.
6. Carefully break the swab shaft at the score line against the side of the tube.
7. Immediately discard the top portion of the swab shaft.
8. Tightly screw the cap onto the tube.
